# Soft phononic crystals with deformation-independent band gaps

**DOI:** 10.1098/rspa.2016.0865

**Published:** 2017-04-05

**Authors:** Pu Zhang, William J. Parnell

**Affiliations:** School of Mathematics, University of Manchester, Oxford Road, Manchester M13 9PL, UK

**Keywords:** phononic crystal, transformation elasticity, hyperelastic, neo-Hookean, semilinear

## Abstract

Soft phononic crystals have the advantages over their stiff counterparts of being flexible and reconfigurable. Normally, the band gaps of soft phononic crystals will be modified after deformation due to both geometric and constitutive nonlinearity. Indeed these are important properties that can be exploited to tune the dynamic properties of the material. However, in some instances, it may be that one wishes to deform the medium while retaining the band gap structure. A special class of soft phononic crystals is described here with band gaps that are independent or almost-independent of the imposed mechanical deformation, which enables the design of phononic crystals with robust performance. This remarkable behaviour originates from transformation elasticity theory, which leaves the wave equation and the eigenfrequencies invariant after deformation. The necessary condition to achieve such a property is that the Lagrangian elasticity tensor of the hyperelastic material should be constant, i.e. independent of deformation. It is demonstrated that incompressible neo-Hookean materials exhibit such a unique property. Semilinear materials also possess this property under special loading conditions. Phononic crystals composed of these two materials are studied theoretically and the predictions of invariance, or the manner in which the response deviates from invariance, are confirmed via numerical simulation.

## 1. Introduction

Phononic crystals (PCs) are periodic structures that can control the propagation of acoustic or elastic waves via wave filtering in specific frequency ranges [1–10]. Potential applications of PCs include waveguides and filters, sensors, redirectivity devices and many more. Early studies focused predominantly on *stiff* PCs, which have negligible deformation under general external loads. Recently, however, attention has switched to the study of *compliant* or *soft* phononic crystals (SPCs) due to their potential for flexibility, tunability and multifunctionality. To date, almost all work on SPCs is related to their tunability, i.e. band gaps are tuned via mechanical deformation or some other mechanism such as an imposed electrical or magnetic field.

A variety of tuning methods using mechanical deformation have been proposed. Simple one-dimensional models were studied initially [11,12] and since then Bertoldi and co-workers have conducted extensive studies on tuning the band gaps of hyperelastic PCs with the aid of structural instability [13–15]. Rudykh & Boyce [16] discovered that the wrinkling of thin stiff layers embedded in a compliant matrix could tune band gaps and more general wave propagation behaviour. Mousanezhad *et al.* [17] designed flexible honeycomb structures with tunable band gaps under compression and buckling. Most of these aforementioned works are related to body waves. Recently, however, Li *et al.* [18] demonstrated that surface wave band gaps can also be tuned by controlling the surface wrinkling patterns of soft materials. In addition to exploiting instability, a number of other methods have been proposed to tune the dynamic behaviour of PCs. Tang *et al.* [19] designed super-stretchable structures with cut hinges to achieve both tunable band gaps and enhanced strength simultaneously. Barnwell *et al.* [20,21] proposed the idea of tuning band gaps of soft crystals by imposing local pre-deformation without inducing global deformation. Galich *et al.* studied band gap tunability in layered SPCs [22]. Magnetic and electrical effects to tune band gaps have also been described [23–27] having the advantage that contact with the structure is not required, although the constituent materials themselves are generally more complex. Significant progress has therefore been made in the design of tunable PCs by employing mechanical deformation and related techniques.

Given that the majority of the work thus far published has centred on the notion that SPCs are employed to *tune* the band gaps of the medium, an interesting question arises as to *whether it is possible to design SPCs with invariant band gaps even after large deformation?* If this is answered in the affirmative, one would be able to design PCs with robust frequency response while maintaining flexibility and multifunctionality. The primary aim of this work therefore is to explore the conditions required to achieve this apparently abnormal behaviour. It will be shown that this remarkable phenomenon is closely related to the transformation elasticity theory used to design elastodynamic cloaks [28–30].

In §2, the notion of band gap invariance in one-dimensional structures is first discussed before proceeding to the general theory for two-dimensional structures in §3. The properties of the required materials are discussed in §4 and simulations illustrating the influence of deformation on wave propagation for such special materials are then described in §§5 and 6. Conclusion and a general summary are given in §7.

## 2. One-dimensional phononic crystals under pre-stretch

**Figure 1.**
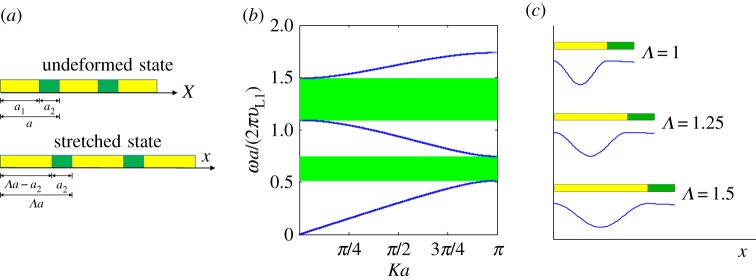
(*a*) Schematic of a one-dimensional soft phononic crystal (SPC) under pre-stretch. The reference state (or undeformed state) is indicated by *Λ*=1, while the stretched state is characterized by the stretch ratio *Λ*. (*b*) Band structure of an SPC with constant Lagrangian stiffness *C*_*L*_. The band structure is independent of the stretch ratio *Λ*. (*c*) A typical wave mode *u*(*x*) with different stretch ratios *Λ*. The wave mode in the compliant layer is in fact uniformly stretched in this case. (Online version in colour.)

In order to illustrate the effect of pre-deformation on the band structure and wave propagation characteristics of an SPC, consider a simple one-dimensional medium as depicted in figure 1*a*.

The unit cell of this PC is composed of a compliant layer and a stiff layer, having initial lengths *a*_1_ and *a*_2_, where the subscript indicates the respective phase of the medium. The initial lattice parameter of the PC is therefore *a*=*a*_1_+*a*_2_. Under uniaxial stretch perpendicular to the faces of the layers, if the elongation of the stiff layer is negligible, the unit cell of the PC deforms to a length *Λa*, where *Λ* is the overall stretch ratio. Either a Lagrangian or Eulerian scheme can be used to describe the deformation and equilibrium of the PC. Assume that the per-unit-length stiffness and mass density are denoted by *C*_*E*_ and *M*_*E*_, respectively, where the subscript ‘E’ or ‘L’ indicates the Eulerian or Lagrangian scheme, respectively. Therefore, whenever the Lagrangian scheme is used, the corresponding stiffness and mass density are obtained as *C*_*L*_=*C*_*E*_/λ and *M*_*L*_=*λM*_*E*_, respectively, where λ is the stretch ratio of any material point. In this case, the wave equation for a linear wave superimposed on the pre-deformation is

2.1 $$C_{L}u_{,XX}=M_{L}u_{,tt}\;\left(\mathrm{Lagrangian}\;\mathrm{scheme}\right)$$

and

2.2 $$C_{E}u_{,xx}=M_{E}u_{,tt}\;\left(\mathrm{Eulerian}\;\mathrm{scheme}\right),$$

where *u* is the displacement, *X* and *x* indicate the reference and spatial coordinates (figure 1*a*), respectively, *t* is time and *f*_,*x*_ indicates the derivative of the function *f* with respect to *x*.

The dispersion relation, i.e. the eigenfrequency *ω*(*K*) as a function of the Lagrangian wavevector *K*, can be readily obtained by assuming a Bloch wave solution

2.3 $$u(X,t)=\tilde{u}(X)\exp[i(KX-\omega t)]$$

and solving the resulting eigenvalue problem, where $\tilde{u}(X)$ is the periodic eigenmode. A variety of methods can be employed to obtain the dispersion curves and wave modes, e.g. the plane wave expansion scheme, transfer matrix method and finite-element method, to name just a few, see [1] for more details. In general, the dispersion curves are strongly dependent on the imposed deformation *Λ* of the structure. Hence, the band structure of an SPC will usually be tuned under pre-stretch. An unusual but intriguing phenomenon would arise when

2.4 $$C_{L}(\lambda )=C_{L}^{0},$$

where $C_{L}^{0}$ is a constant that is independent of the stretch λ. In this case, the Lagrangian wave equation is independent of the stretch, and, therefore, the wave dispersion relation *ω*(*K*) *is independent of deformation*. Therefore, the band structure of the PC will be independent of the uniaxial stretch if equation (2.4) is satisfied.

The longitudinal wave speed is $v_{L}=\sqrt{C_{L}/M_{L}}$ and $v_{E}=\sqrt{C_{E}/M_{E}}$ for the Lagrangian and Eulerian scheme, respectively. The Lagrangian wave speed *v*_*L*_ is independent of the stretch if *C*_*L*_ is a constant. In this case, the Eulerian wave speed *v*_*E*_=*λv*_*L*_ is proportional to the stretch ratio λ, i.e.

2.5 $$v_{E}\propto \lambda .$$

Therefore, as illustrated in figure 1*c*, the wavefront will arrive at the same material point during a given time interval, no matter how large the pre-stretch ratio is. By contrast, the spatial wave speed is proportional to the stretch ratio according to equation (2.5).

An example is provided in order to illustrate the band structure and eigenmodes of an SPC with deformation-independent band gaps. The geometric and material constants are taken as *a*_1_=2*a*/3, *a*_2_=*a*/3, *v*_*L*2_=10*v*_*L*1_, respectively. As illustrated in figure 1*b*, the band structure, if plotted in the Lagrangian scheme as *ω*-*K*, is independent of the overall stretch ratio *Λ*. However, the wave mode *u*(*x*) will be stretched in the spatial domain, as shown in figure 1*c*, which depicts the wave mode for one unit cell (*K*=0.315/*a*, *ω*=9.425*v*_*L*1_/*a*). Therefore, the amplitude, phase and shape of the waves are actually retained during the deformation due to the constant Lagrangian wave speed *v*_*L*_ in the constituent materials. In fact, the material points do not sense any changes of their own motion or neighbourhood in this case. This is the essential reason causing such an intriguing phenomenon with deformation-independent band gaps in one-dimensional SPCs. One could go on to consider the type of material required to produce this type of effect in this one-dimensional scenario. However, given that the purpose of this section was primarily to illustrate the notion of invariance this is bypassed and more complex, realistic configurations are now considered.

## 3. Soft phononic crystals in higher dimensions

In §2, it has been shown that the band gap structure of an SPC in one dimension is independent of stretch and deformation if the Lagrangian wave speed is constant. Does this phenomenon exist in higher dimensions? In order to address this question, the fundamental theory for SPCs will be introduced in this section and a necessary condition for deformation-independent band structures will be discussed.

### (a) Soft phononic crystals with pre-deformation

**Figure 2.**
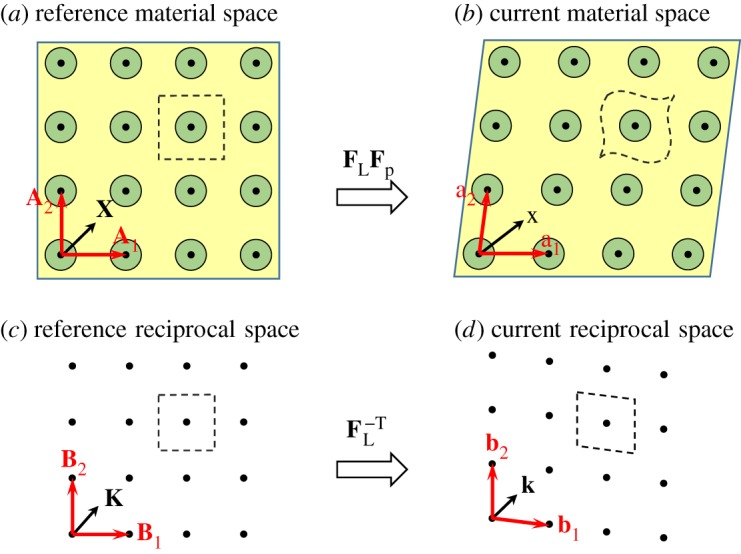
Schematic of the material configurations and reciprocal spaces of a soft PC under affine lattice deformation. The boundaries of undeformed and deformed unit cells are indicated by dashed curves. The periodic lattice in the reference configuration in (*a*) is mapped to the current configuration in (*b*) via the affine transformation **F**_*L*_ with periodic deformation **F**_*p*_. It is noted (and depicted in (*b*)), however, that a general point in the medium will not transform in this manner. The reciprocal space transforms via the affine mapping **F**^−*T*^_*L*_ from (*c*) to (*d*). (Online version in colour.)

As shown in figure 2, a two-dimensional PC is considered in this work, but the proposed theory and mechanism also applies for SPCs in general. Although more general designs are possible as will be described below, consider for now the case when the SPC comprises stiff cylinders embedded on a square lattice in a compliant matrix. Therefore, it is possible to deform the PC in figure 2*a* in a finite manner, into the current configuration as depicted in figure 2*b* via the global affine lattice deformation. Correspondingly, the lattice vectors **A**_*i*_ (*i*=1,2,3) in the reference material space will transform to lattice vectors **a**_*i*_ in the current material space via

3.1 $${\text{a}}_{i}={\text{F}}_{L}{\text{A}}_{i},$$

where **F**_*L*_ designates the affine deformation gradient [31–33] of the lattice points. Note that the reference position **X** and current position **x** of an arbitrary material point will not in general obey the affine transformation, i.e. **x**≠**F**_*L*_**X** as illustrated in figure 2*a*,*b*, due to the periodic non-affine deformation **F**_*p*_(**X**) within a unit cell and hence the total deformation gradient [33] is **F**(**X**)=**F**_*L*_**F**_*p*_(**X**).

Corresponding to the material spaces, the reference reciprocal space (figure 2*c*) and current reciprocal space (figure 2*d*) can also be related by a mapping. More specifically, the mapping between these two spaces is affine since the reciprocal space is derived from the lattice points in the reference material space, regardless of the constituent materials and interior geometry of the material unit cell. The lattice vectors in the reference and current reciprocal spaces are denoted as (**B**_1_,**B**_2_,**B**_3_) and (**b**_1_,**b**_2_,**b**_3_), respectively, which are defined by **A**_*i*_⋅**B**_*j*_=**a**_*i*_⋅**b**_*j*_≡2*πδ*_*ij*_, where *δ*_*ij*_ is the Kronecker delta tensor. Therefore, the affine mapping between the two reciprocal spaces is derived as

3.2 $${\text{b}}_{i}={\text{F}}_{L}^{-T}{\text{B}}_{i}\;(i=1,2,3)$$

due to the fact that

3.3 $$\begin{array}{rl} {\text{A}}_{i}\cdot {\text{B}}_{j} & ={\text{a}}_{i}\cdot {\text{b}}_{j}\\ & ={\text{F}}_{L}{\text{A}}_{i}\cdot {\text{b}}_{j}\\ & ={\text{A}}_{i}\cdot {\text{F}}_{L}^{T}{\text{b}}_{j}. \end{array}$$

Consequently, one can readily obtain the mapping between the wavevectors in the two reciprocal spaces. By denoting the wavevectors in the reference and current reciprocal spaces as **K** and **k**, respectively, they can be related via the simple relationship

3.4 $$\text{k}={\text{F}}_{L}^{-T}\text{K}.$$

### (b) Incremental dynamic equation

Linear elastic wave propagation in SPCs with large pre-deformation is described by incremental elastodynamic equations [34], derived via what is often called the theory of *small-on-large*. This theory has been widely used to study elastic wave propagation and scattering in soft materials with pre-deformation [13,14,16,18,20,22,28,29,35,36]. There are two equivalent sets of incremental equations that can be employed to study wave propagation in the pre-stressed state: the *Lagrangian* and *Eulerian* forms [34]. The former specifies equations in the reference (undeformed) material space while the latter specifies the current (deformed) material space. The Lagrangian form is often convenient since boundary conditions can be imposed in a more straightforward manner. The Lagrangian form is adopted in this paper; this gives

3.5 $$A_{ijk\ell ,j}u_{k,\ell }+A_{ijk\ell }u_{k,j\ell }=\rho_{0}u_{i,tt},$$

where *f*_,*i*_=∂*f*/∂*X*_*i*_, *u*_*i*_ is the *i*th component of displacement **u**, resolved along Cartesian coordinates, *ρ*_0_ is the material density in its undeformed state and A is the incremental elasticity tensor (ET). This ET is usually a function of the deformation gradient **F** and its components are defined by

3.6 $$A_{ijk\ell }=\frac{\partial^{2}W}{\partial F_{ij}\partial F_{k\ell }},$$

where *W*(**F**) is the strain energy function of the hyperelastic material under deformation [34,37].

The Bloch wave solution is taken as $\text{u}(\text{X},t)=\tilde{\text{u}}(\text{X})\exp(-i\omega t)$, where $\tilde{\text{u}}(\text{X})$ is known as the Bloch wave mode. The Bloch boundary condition [13] can be formulated in either the reference or current reciprocal space, depending on the convenience of the calculation. With regard to the former, Bloch's theorem requires that the displacement fields $\tilde{\text{u}}(\text{X})$ satisfy

3.7 $$\tilde{\text{u}}(\text{X}+{\text{A}}_{i})=\tilde{\text{u}}(\text{X})\exp(i\text{K}\cdot {\text{A}}_{i})$$

due to the translational invariance of the PCs. Note that similar formulae can be expressed in the current material/reciprocal space as well. The dispersion curves *ω*(**K**) of the PCs are actually obtained by solving the eigenvalue problem for equation (3.5) by prescribing the Bloch condition in equation (3.7) for a given wavevector **K**.

### (c) Transformation elasticity and eigenfrequency invariance

Transformation elasticity theory [29] provides a scenario where the incremental wave equation (3.5) is invariant to pre-deformation. This is achieved when the ET is independent of the deformation, i.e.

3.8 $$A(\text{F})=A^{0},$$

where $A^{0}$ is a tensor with constant components that is independent of **F**. Similar to the one-dimensional example, an intriguing phenomenon arises when equation (3.8) is satisfied. In this case, the incremental equations (3.5) further simplify to $A_{ijk\ell }^{0}u_{k,j\ell }=\rho_{0}{\ddot{u}}_{i}$, i.e. they are independent of the imposed large deformation. If all phases of a PC satisfy this condition, one would expect that the eigenfrequencies $\omega_{n}(\text{K})\;(n=1,2,\ldots ,\infty )$ are independent of the deformation, no matter how large it is. Consequently, the band gaps would also be invariant with respect to the mechanical deformation. This mechanism is valid for SPCs in any dimension. Therefore, the key issue to the design of SPCs with deformation-independent band gaps is to guarantee that the ET A is constant (invariant of deformation) for all phases. Three types of two-phase designs are proposed: (i) a solid–solid PC with constant ET for both phases; (ii) a solid–void PC with constant ET for the matrix phase; (iii) a solid–solid PC where the compliant matrix has a constant ET and the inclusion phase is stiff enough so that its ET is deemed to remain constant during deformation. In this work, attention is focused predominantly on materials of type (iii) although an example of type (ii) is also considered in §6c.

## 4. Required hyperelastic strain energy functions

### (a) Materials with constant elasticity tensor

What kind of hyperelastic material will exhibit an ET A with constant components? One should expect such materials to be very rare. The mathematical forms of the strain energy function that are required to attain constant ETs shall be derived. Attention shall be restricted to isotropic materials, either compressible or incompressible. According to Ogden [38], an alternative formulation for the ET A in equation (3.6) is

4.1 $$A_{ijk\ell }=\delta_{ik}S_{j\ell }+F_{im}C_{mjn\ell }F_{kn},$$

where **S**=∂*W*/∂**E** is the second Piola–Kirchhoff stress, $C=\partial^{2}W/\partial \text{E}\partial \text{E}$ is the instantaneous modulus, and **E**=(**F**^*T*^**F**−**I**)/2 is the Green–Lagrange strain tensor. The first term on the right-hand side of (4.2) depends on the material stretch only, while the second term depends on both material stretch *and* rotation. Therefore, in order to obtain a constant ET $A^{0}$, for general deformations, the second Piola–Kirchhoff stress **S** should be constant and the instantaneous modulus C must always be identically zero. Hence, the strain energy function *W* must be a linear function of the first invariant of **E**, i.e.

4.2 $$W=\mu_{0}\;\text{tr}\;\text{E},$$

where *μ*_0_ is the shear modulus and **E**=*E*_*ii*_. The strain energy function in equation (4.2) will inevitably result in a hydrostatic stress term in the undeformed state and it does not appear possible to modify this form for compressible materials in a manner consistent with the imposition that A remains constant. For *incompressible* materials however a Lagrange multiplier term *p* can be added, i.e. the strain energy function becomes

4.3 $$W=\mu_{0}\;\text{tr}\;\text{E}-p(\;J-1),$$

where J=detF→1 for incompressible materials. We see that this form is consistent with an incompressible neo-Hookean material. The ET A of neo-Hookean materials can be derived from equation (4.2), as

4.4 $$A_{ijk\ell }=\mu_{0}\delta_{ik}\delta_{j\ell }.$$

The conclusion here then is that an incompressible neo-Hookean material is the only material with a constant ET that is independent of the finite deformation and is consistent with the usual restrictions on strain energy functions for hyperelastic materials.

### (b) Materials with almost-constant elasticity tensors

As has been illustrated above, it is very challenging to find materials with deformation-independent elastic tensors when subjected to general deformations. Therefore, attention is now restricted to materials with almost-constant ETs or where specific modes of deformation permit invariance of specific wave types. The explicit form of A is usually lengthy, although a very compact form for isotropic materials is straightforwardly derived by expressing the tensor with respect to the principal axes of the right stretch tensor **U** or left stretch tensor **V**. Denoting such a representation as $\hat{A}$, its non-zero components are given as [34,38]

4.5 $$\begin{array}{rl} {\hat{A}}_{iijj} & =W_{ij}, \end{array}$$

4.6 $$\begin{array}{rl} {\hat{A}}_{ijij}-{\hat{A}}_{ijji} & =\frac{W_{i}+W_{j}}{\lambda_{i}+\lambda_{j}}\;i\neq j, \end{array}$$

4.7 $$\begin{array}{rl} {\hat{A}}_{ijij}+{\hat{A}}_{ijji} & =\frac{W_{j}-W_{i}}{\lambda_{j}-\lambda_{i}}\;i\neq j,\lambda_{i}\neq \lambda_{j} \end{array}$$

4.8 $$\begin{array}{rl} \text{and}\;{\hat{A}}_{ijij}+{\hat{A}}_{ijji} & =W_{jj}-W_{ij}\;i\neq j,\lambda_{i}=\lambda_{j}. \end{array}$$

Here *W*_*i*_=∂*W*/∂λ_*i*_,*W*_*ij*_=∂^2^*W*/∂λ_*i*_∂λ_*j*_ and, importantly, the summation rule does not apply to repeated indices. Note that the ET A can be obtained by applying coordinate rotations to $\hat{A}$, and it is important to stress that for general deformations the principal axes will vary with position. Full invariance is therefore not possible in general due to the presence of rotations, although if there is a homogeneous set of principal axes, invariance *is* a possibility.

The formulae in equations (4.5)–(4.8) imply that for invariance, the strain energy function *W* should be a quadratic polynomial function of the principal stretches λ_1_,λ_2_,λ_3_ at least. Any higher powers will in general lead to stretch-dependence. In addition, the constitutive theory requires that *W* be expressed in terms of the invariants of the right stretch tensor **U** for isotropic hyperelastic materials. Hence, the following polynomial form of strain energy function is chosen

4.9 $$W=c_{0}+c_{1}i_{1}+c_{2}i_{1}^{2}+c_{3}i_{2}$$

for compressible materials, where *c*_*i*_ (*i*=0,1,2,3) are unknown coefficients to be determined, and *i*_1_=*tr* **U**, *i*_2_=(*tr*^2^**U**−*tr* **U**^2^)/2 are the first two invariants of **U**. Two of the coefficients *c*_*i*_ can be eliminated by using the energy-free (*W*=0) and stress-free (∂*W*/∂λ_*i*_=0) conditions in the undeformed state (λ_*i*_=1). It follows that the strain energy function required is the semilinear form, i.e. [29]

4.10 $$W=\frac{1}{2}\lambda_{0}{\left(i_{1}-3\right)}^{2}+\mu_{0}[{\left(i_{1}-1\right)}^{2}-2(i_{2}-1)],$$

where λ_0_ and *μ*_0_ are the Lamé constant and shear modulus, respectively. The first Piola–Kirchhoff stress **P** of the semilinear model is derived from the relation **R**^*T*^**P**=∂*W*/∂**U** [38], as

4.11 $$\text{P}=2\mu_{0}\;\text{F}+[\lambda_{0}(i_{1}-3)-2\mu_{0}]\text{R},$$

where **R** is the rotation tensor of **F**.

The ET of the semilinear model is derived by substituting equation (4.10) in equations (4.5)–(4.8), as

4.12 $$\begin{array}{rl} {\hat{A}}_{iijj} & =\lambda_{0}+2\mu_{0}\delta_{ij}, \end{array}$$

4.13 $$\begin{array}{rl} {\hat{A}}_{ijij}-{\hat{A}}_{ijji} & =\frac{2\lambda_{0}(i_{1}-3)-4\mu_{0}}{\lambda_{i}+\lambda_{j}}+2\mu_{0},\;i\neq j \end{array}$$

4.14 $$\begin{array}{rl} \text{and}\;{\hat{A}}_{ijij}+{\hat{A}}_{ijji} & =2\mu_{0},\;i\neq j. \end{array}$$

Equation (4.13) indicates that, as expected, for general deformations, the stretch deformation will affect some of the components of the ET, while the major terms ${\hat{A}}_{iijj}$ in equation (4.12) will always be constant. Hence, the semilinear model has an almost-constant ET.

For special cases, the components can be further simplified. A special case is the situation when *W*_*i*_+*W*_*j*_=0 which renders the right-hand side of (4.13) zero. As was determined in equation (4.14) of [29], for in-plane incremental displacements superposed on a finite homogeneous deformation, the following condition achieves this:

4.15 $$\lambda_{3}=1-\frac{1}{2\nu_{0}}(\lambda_{1}+\lambda_{2}-2),$$

where *ν*_0_ is Poisson's ratio. In this case then, the ET related to the *in-plane* deformation degenerates to a constant tensor, i.e.

4.16 $$A_{ijk\ell }=\lambda_{0}\delta_{ij}\delta_{k\ell }+\mu_{0}(\delta_{ik}\delta_{j\ell }+\delta_{i\ell }\delta_{jk})\;(i,j=1,2).$$

In other words, the in-plane ET is dependent neither on the in-plane stretch (λ_1_,λ_2_) nor the in-plane rotation. This is therefore a special case when the semilinear material exhibits an invariant ET and this will be exploited to design an invariant SPC of type (ii) as defined in §3c.

A straightforward case to consider, but which will inevitably lead to stretch-dependence of A is the plane strain case (λ_3_=1). In this situation, equation (4.13) reduces to the following form:

4.17 $${\hat{A}}_{ijij}-{\hat{A}}_{ijji}=\frac{2(\lambda_{0}+\mu_{0})(\lambda_{i}+\lambda_{j}-2)}{\lambda_{i}+\lambda_{j}}\;(i,j=1,2;\;i\neq j).$$

Therefore, the term λ_0_+*μ*_0_ in equation (4.17) should be minimized to reduce the effect of stretch on A. This is equivalent to reducing Poisson's ratio *ν*_0_ because λ_0_+*μ*_0_=*μ*_0_/(1−2*ν*_0_). If Poisson's ratio $\nu_{0}\to 0.5$, the Lamé constant $\lambda_{0}\to \infty$, which will cause significant stretch-dependence of the components of A.

In addition to the effect of stretch, as described above, the transformed ET A will also depend on rotation in general.

## 5. Wave speed invariance

In §2, it was shown that band gap invariance in one-dimensional SPCs is closely related to Lagrangian wave speed invariance. Therefore, the effect of pre-deformation on the wave speed of plane waves in *homogeneous* neo-Hookean and semilinear materials is studied in this section. A homogeneous sample is stretched uniformly along the *X*_1_ direction with stretch ratio λ_1_=*Λ*. The medium is constrained along the *X*_3_ direction, i.e. λ_3_=1 is imposed. The condition associated with stretch in the *X*_2_ direction will be chosen for each example to be considered. For neo-Hookean materials, the focus shall be on the horizontally polarized (SH mode, polarized in the *X*_3_ direction) and vertically polarized (SV mode, polarized in the *X*_2_ direction) shear waves, while for the semilinear material the additional longitudinal (P mode) wave is considered.

First, consider shear waves (SV and SH modes) propagating in the *X*_1_ direction in an incompressible neo-Hookean material under uniform pre-stretch. The stretch ratios are taken to be λ_1_=*Λ*,λ_2_=*Λ*^−1^,λ_3_=1 so the deformation is isochoric. The Lagrangian wave speed for plane waves can be derived from equation (3.5) as

5.1 $$v_{Li}=\sqrt{\frac{A_{i1i1}}{\rho_{0}}}\;(i=1,2,3),$$

where the subscript *i*=1,2,3 for the P, SV and SH wave mode, respectively. The ET components can be derived from equation (4.4), as $A_{2121}=A_{3131}=\mu_{0}$, regardless of the pre-deformation. Therefore, the Lagrangian wave speed of the shear waves is also constant as $v_{L2}=v_{L3}=\sqrt{\mu_{0}/\rho_{0}}$, while the corresponding Eulerian wave speed is $v_{E2}=v_{E3}=\Lambda \sqrt{\mu_{0}/\rho_{0}}$, proportional to the stretch ratio. The shear wave speed invariance of the incompressible neo-Hookean material means that it is a possible material that could be used as an invariant SPC when interest is focused only on shear waves, although of course in general there will be coupling to compressional waves when the medium is inhomogeneous.

The analysis of the semilinear material is more complicated than the neo-Hookean material. Suppose that the medium is stress-free in the *X*_2_ direction, i.e. imposing *P*_22_=0, so that the pre-stretch ratios can be shown to be (using (4.11))

5.2 $$\lambda_{1}=\Lambda ,\;\lambda_{2}=\frac{1-\Lambda \nu_{0}}{1-\nu_{0}},\;\lambda_{3}=1.$$

The ET components are derived from equations (4.12)–(4.14), as

5.3 $$\begin{array}{rl} A_{1111} & =\lambda_{0}+2\mu_{0}, \end{array}$$

5.4 $$\begin{array}{rl} A_{2121} & =\frac{2\Lambda (1-\nu_{0})\mu_{0}}{1+\Lambda (1-2\nu_{0})} \end{array}$$

5.5 $$\begin{array}{rl} \text{and}\;A_{3131} & =\frac{2(\Lambda -\nu_{0})\mu_{0}}{\left(1+\Lambda )(1-\nu_{0}\right)}. \end{array}$$

**Figure 3.**
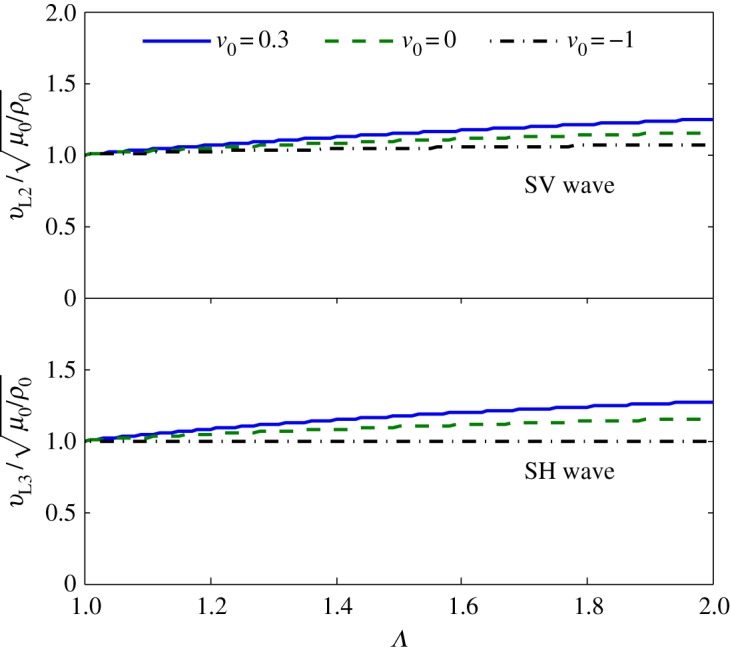
Influence of the stretch ratio *Λ* on the Lagrangian wave speed of shear waves in semilinear materials with different Poisson's ratio *ν*_0_ under the specified pre-deformation in equation (5.2). (Online version in colour.)

The wave speeds in the homogeneous material can then be readily derived from equation (5.1). The Lagrangian wave speed *v*_*L*1_ of the P wave is therefore independent of the stretch ratio *Λ*, while the shear wave speeds *v*_*L*2_ or *v*_*L*3_ of the shear wave modes depend on the stretch ratio *Λ*. As shown in figure 3, the Lagrangian wave speeds of the SV and SH waves in the semilinear material are slightly affected by the stretching ratio *Λ*, and the stretch-dependent effect is reduced when Poisson's ratio *ν*_0_ decreases. In addition, the SH wave speed is found to be independent of stretch when Poisson's ratio *ν*_0_=−1, which can be observed in figure 3 and verified from equation (5.5).

Now consider a third case; the special scenario associated with the imposition of equation (4.15), which gives the invariant in-plane ET as in equation (4.16). The stretch ratios are taken as λ_1_=*Λ*, λ_2_=2−*Λ* and λ_3_=1. The Lagrangian wave speeds of the in-plane P-SV waves are not affected by the pre-deformation. However, the components of the ET corresponding to SH wave propagation will be affected by the pre-stress. Suppose that an SH wave propagates in a direction defined by an angle *θ* subtended from the *X*_1_ axis. The corresponding Lagrangian wave speed is then

5.6 $$\begin{array}{rl} v_{L}^{\mathrm{SH}} & =\sqrt{\frac{A_{3131}\cos^{2}\theta +A_{3232}\sin^{2}\theta }{\rho_{0}}} \end{array}$$

5.7 $$\begin{array}{rl} & =\sqrt{\frac{\mu_{0}}{\rho_{0}}}\sqrt{\frac{2\Lambda }{1+\Lambda }+\frac{4(\Lambda -1)}{\Lambda^{2}-2\Lambda -3}\sin^{2}\theta }. \end{array}$$

**Figure 4.**
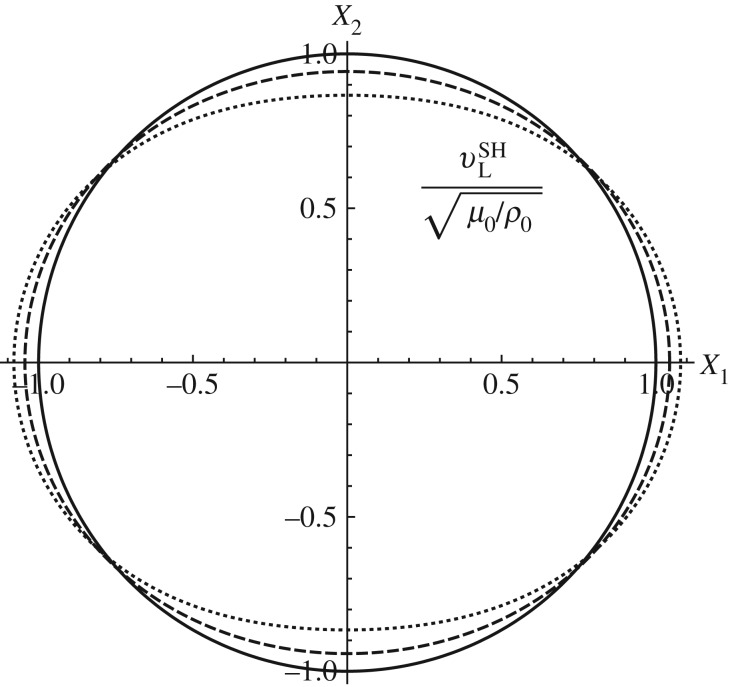
Polar plot of the Lagrangian wave speed of SH waves in semilinear materials with pre-deformation λ_1_=*Λ*, λ_2_=2−*Λ* and λ_3_=1. The solid, dashed and dotted curves represent *Λ*=1,1.2,1.4, respectively. For this deformation in semilinear materials, in-plane waves possess invariant Lagrangian wave speeds.

The above equation indicates that the Lagrangian wave speed of the SH waves will be anisotropic depending on the pre-stretch ratio *Λ*. This effect of induced anisotropy for the SH wave is illustrated in figure 4.

To summarize, the analysis above indicates that the Lagrangian wave speed of shear waves in an incompressible neo-Hookean material is independent of the deformation. Hence, the neo-Hookean material has potential in designing PCs with deformation-independent band gaps for shear wave modes. On the other hand, the semilinear material cannot exhibit such total invariant behaviour. Having said this, the Lagrangian wave speed of plane waves in a deformed semilinear material are unaffected (for P waves) or slightly affected (for SV and SH waves) by pre-deformation. Hence, one should expect that band gaps in semilinear PCs can be almost-independent of the pre-deformation in general. For a specific case, the semilinear material may exhibit a constant in-plane ET for the special pre-deformation mode as in equation (4.15). This gives rise to the possibility of designing SPCs with deformation-independent in-plane (P/SV) band gaps. These hypotheses will be verified in the next section where wave propagation in various configurations of SPCs will be simulated numerically.

## 6. Simulation and results

### (a) Simulation model

Based on the above analysis, a PC comprising an incompressible neo-Hookean material will have deformation-independent band gaps for shear waves, while one composed of a semilinear material will have band gaps that are almost-invariant for all wave modes. Full in-plane invariance of the semilinear material is achievable for the special deformation case given in equation (4.15), which leads to invariant P-SV band gaps. The effect is illustrated below by considering two examples of SPCs of type (iii) and one example of type (ii) as classified at the end of §3c. An infinitely thick PC (figure 2*a*) is considered with cylinders organized on a square lattice. For the medium of type (iii), the inclusion phase is stiff enough so that its ET is independent of deformation since the induced strain is infinitesimal. For the type (ii) material the inclusion is a void. The matrix phase is neo-Hookean in the first example, and semilinear in subsequent examples. The phononic band structures are calculated by using the finite-element package ABAQUS (v. 6.13-1) and adopting the method proposed by Åberg & Gudmundson [39,13]. One layer of linear brick elements C3D8R are built for the simulation model and the Bloch condition is imposed on the boundaries of the unit cell. A unique feature of this model is that the in-plane mode (P-SV waves) and antiplane mode (SH waves) can be decoupled. The band structures are therefore plotted separately for these two scenarios.

### (b) Phononic crystals of neo-Hookean type

For the first example, a compressible neo-Hookean material is employed so that both the P-SV and SH waves can propagate, although the latter is of specific interest. The corresponding strain energy function is taken as

**Figure 5.**
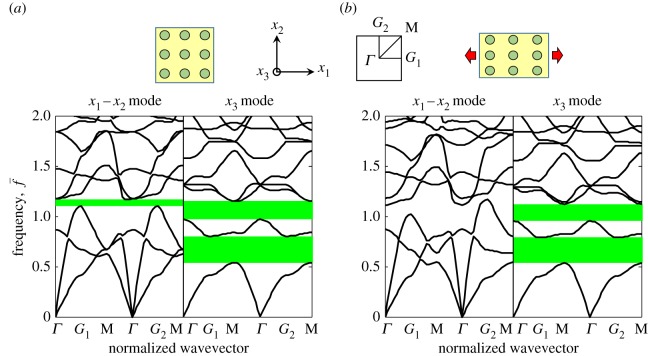
Band structures of an SPC with stiff elastic cylinders embedded in a compressible neo-Hookean matrix, in the case of the (*a*) undeformed state, (*b*) deformed state under uniaxial tension of the lattice. Band gap regions are highlighted in green.Although the antiplane wave band gap regions remain fixed during deformation, those associated with the in-plane waves will be deformation dependent for this material type, as predicted by the theory. (Online version in colour.)

6.1 $$W=\frac{1}{2}\mu_{m}(J^{-2/3}I_{1}-3)+\frac{1}{2}\kappa_{m}{\left(J-1\right)}^{2}$$

where *I*_1_= **U**^2^, *J*= det **F**, the shear modulus *μ*_*m*_=25.9 MPa, bulk modulus *κ*_*m*_=10*μ*_*m*_ and density *ρ*_*m*_=1000 kg m^−3^. The cylindrical inclusions are taken to be aluminium with shear modulus *μ*_*c*_=25.9 GPa, Poisson's ratio *ν*_*c*_=0.33, and density *ρ*_*c*_=2700 kg m^−3^. Additionally in this example, uniaxial stretching (*ϵ*=15% elongation with the lateral sides left free, where *ϵ* is the engineering strain calculated over the unit cell) is applied to the PC, as illustrated in figure 5*b*. For the stretch deformation, the lattice deformation gradient components are *F*_*L*11_=1+*ϵ*, *F*_*L*12_=*F*_*L*21_=0 and *F*_*L*22_ is determined by the stress-free condition on the lateral sides. The band structures for the undeformed and stretched PCs are shown in figure 5*a*,*b*, respectively, with dispersion curves *ω*_*n*_(**K**) calculated along the path *Γ*-*G*_1_-M-*Γ*-*G*_2_-M in the Brillouin zone (reference reciprocal space). The dimensionless frequency is defined as

6.2 $$\bar{f}=\frac{\omega a}{2\pi \sqrt{\mu_{m}/\rho_{m}}},$$

where *ω* is the angular frequency and *a*=0.1 *m* is the characteristic length of the unit cell. Additionally, the radius of the cylindrical inclusions is 0.03 m. By comparing the band gaps in figure 5, it is observed that the single-band gap (located in the vicinity of $\bar{f}=1.1$) of the in-plane wave modes (labelled as the *x*_1_−*x*_2_ mode) disappears after stretching. This is because in this case the ET components associated with in-plane waves are deformation-dependent. The stretch deformation induces symmetry breaking of the Brillouin zone so that the *G*_1_ and *G*_2_ points are no longer equivalent. This is seen by considering the third band *ω*_3_ associated with in-plane wave modes in figure 5*b*. By contrast, the position of the two band-gaps associated with antiplane waves (the *x*_3_ mode) remain unchanged after stretching since the neo-Hookean material with strain energy function defined in (6.1) behaves very similar to the invariant incompressible medium in response to antiplane shear waves. This confirms the result associated with neo-Hookean media derived theoretically above. Interestingly, invariance of antiplane band gaps has previously been observed in a neo-Hookean SPC [13], but the origin of this phenomenon has not been explained until now. The neo-Hookean material retains the total invariance of transformation elasticity [29]. Given that the antiplane band gaps of the neo-Hookean-type PCs are completely invariant to arbitrary pre-deformation, no further deformation examples for materials of this type will be discussed. Instead attention will focus on the possibility of invariance for in-plane waves.

### (c) Phononic crystals of semilinear type

**Figure 6.**
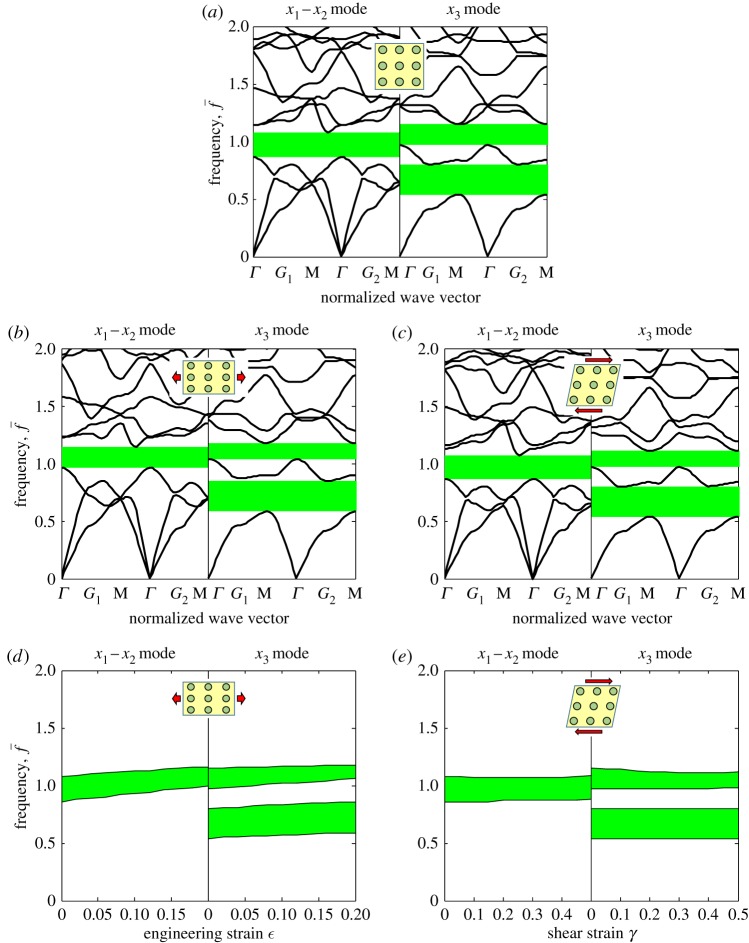
Band structures of an SPC with stiff elastic cylinders embedded in a semilinear hyperelastic matrix, in the case of the (*a*) undeformed state, (*b*) deformed state under uniaxial stretch and (*c*) deformed state under shear deformation. The influence of pre-deformation modes on the band gap frequencies is illustrated in (*d*) and (*e*) for the stretching and shearing deformation cases, respectively. (Online version in colour.)

Consider an SPC with semilinear hyperelastic matrix medium with Lamé constant λ_*m*_=50.4 MPa, shear modulus *μ*_*m*_=25.9 MPa and density *ρ*_*m*_=1000 kg m^−3^. The material configuration is the same as for the neo-Hookean material example considered in the previous section (aluminium cylinders), except that now the compliant matrix is semilinear. Because the semilinear medium is not completely invariant to deformation, two different deformation modes, stretch and shear, are considered for this example. The resulting band structures of the semilinear SPC are shown in figure 6 in the undeformed and deformed cases. For the shear deformation, the lattice deformation gradient components are *F*_*L*12_=*γ*, *F*_*L*21_=0, and *F*_*L*11_ and *F*_*L*22_ are determined from the free normal stress conditions on the four sides.

The ET A associated with semilinear materials is only slightly affected by the pre-deformation. Consequently, although the band structures of the SPCs will be affected by pre-stress, the expectation is that the effect will be weak. This is verified with the numerical simulations carried out here. As shown in figure 6*a*, only one band gap is observed for in-plane waves while two band gaps are determined for antiplane waves in the undeformed state. In the deformed state, the band gaps are slightly tuned, as shown in figure 6*b* for the stretching case (*ϵ*=0.15) and figure 6*c* for the shearing case (*γ*=0.3), respectively. The dependence of the pre-deformation on the band gaps is more clearly shown in figure 6*d*,*e* for the stretch and shear deformation cases, respectively. It is observed that the stretch deformation will affect the band gaps slightly, while the influence of the shear deformation on the band gaps is negligible. One explanation for this difference is that the maximum material stretch in the case of shearing is less than that in the case of uniaxial stretching. It is thus concluded that the band gaps in semilinear SPCs are slightly dependent on the pre-deformation and the influence also depends on the deformation mode considered. The advantage of using materials of semilinear type in SPCs then is that they exhibit relatively robust band gaps for both P-SV and SH waves. This more general case has broader significance and potential applications than using a neo-Hookean material, for which SPCs exhibit invariance only for the antiplane wave mode.

Another interesting phenomenon observed in figure 6 is the wave mode degeneracy at the high symmetry point M right above the second band gap of the *x*_3_ modes. It is found that the two dispersion curve branches, which originally coincide at point M in figure 6*a*, become separated in the corresponding band structures in figure 6*b*,*c*. This is actually induced by the symmetry breaking of the PC once the square lattice is distorted due to the deformation [33,40], e.g. the fourfold rotation and reflection symmetries may not be preserved in the deformed configuration. In theory, this kind of degeneracy change should not occur for an SPC comprising only neo-Hookean phases due to the invariance of the wave equations for the *x*_3_ modes. However, these two branches are slightly separated at point M in figure 5*b* due to the existence of the stiff elastic cylinders. Detailed analysis of the symmetry breaking is quite complicated and will be discussed in future work.

**Figure 7.**
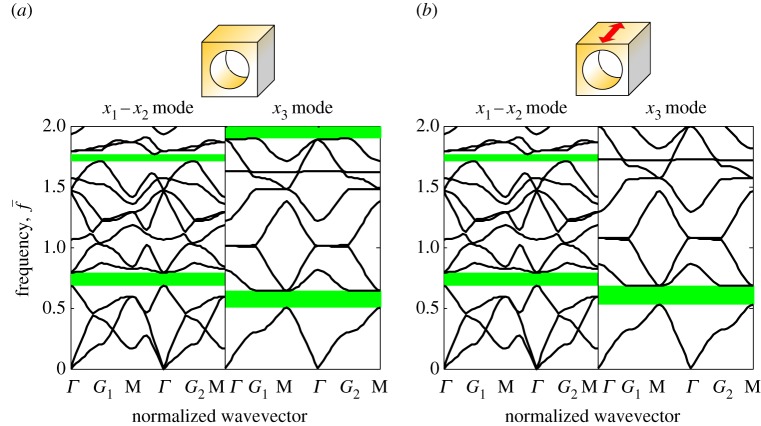
Band structures of an SPC stretched along the *X*_3_ direction. The lateral sides (*X*_1_ and *X*_2_) are kept load-free. (*a*) Undeformed PC.(*b*) PC with pre-stretch λ_3_=1.2. The red arrow indicates the direction of stretch. (Online version in colour.)

The final SPC to be considered is a material of type (ii), i.e. solid–void, with semilinear matrix. Fully invariant P-SV band gaps are possible when the medium is stretched in the *X*_3_ direction (the direction of the axes of the cylindrical voids) while the other two directions are left to be load-free. In this case, the in-plane deformation is homogeneous everywhere (λ_1_=λ_2_=1+*ν*_*m*_−*ν*_*m*_λ_3_) and it can be verified that equation (4.15) is satisfied automatically. Therefore, the in-plane ET components are always constant (invariant) as shown in equation (4.16), while the ET components related to SH waves will depend on the axial stretch λ_3_. Hence, while one should expect that the P-SV band gaps are invariant, the SH band gaps will be tuned under pre-deformation. The simulation results are shown in figure 7, which includes the band structures of the undeformed and pre-stretched SPCs. It is observed that the band structures of the P-SV wave modes are indeed independent of the axial stretch, despite the geometrical deformation, while the band gaps of antiplane waves are affected by the pre-deformation.

In summary, a material of type (iii) (neo-Hookean matrix and aluminium cylindrical inclusions) has been shown to exhibit invariant band gaps for SH waves (for arbitrary deformations), whereas a medium of type (ii) (semilinear matrix with cylindrical voids) exhibits invariant band gaps for P-SV waves (under axial stretch only).

## 7. Conclusion

It has been demonstrated here that it is theoretically possible to design SPCs with deformation-independent band gaps or band gaps that are close to being deformation-independent. This was achieved by appealing to hyperelastic transformation theory. This apparently unusual phenomenon has particular utility in engineering applications requiring both robust frequency response and structural flexibility. In order to achieve this non-trivial property, the key issue is to find materials with deformation-independent ETs so that the Lagrangian-formulated incremental equations possess the property of being invariant to deformation, which is the key notion of transformation elasticity theory. It has been proved that only incompressible neo-Hookean materials satisfy such a constraint exactly (i.e. under all deformations). Semilinear hyperelastic materials exhibit almost-constant ETs for the deformation modes considered here and in-plane invariance for specific materials under axial stretch. It was indicated that the invariance of the band gaps is associated with the invariance of the Lagrangian wave speeds in the medium, which are derived from transformation elasticity. Numerical simulations illustrate that a specified SPC with semilinear hyperelastic matrix and aluminium stiff cylindrical inclusions does indeed have band gaps that are almost-deformation-independent for wave modes in the frequency ranges considered. An example that considered the same cylindrical inclusions in an almost-incompressible neo-Hookean medium on the other hand illustrated that only SH wave band gaps are deformation independent, in accordance with the theoretical predictions. An example involving a solid–void PC of the semilinear type illustrated that deformation-independent P-SV band gaps are possible, but only when the structure is stretched axially. This can be thought of as the counterpart to the invariant SH band gap case for neo-Hookean materials, although while the neo-Hookean case holds for arbitrary deformation the semilinear SPC is only fully invariant under axial stretch. Although the deformation-independence is verified for the Bragg-type band gaps in this work, other types of band gaps, e.g. the one caused by local resonance, are expected to be deformation-independent as long as the Lagrangian-formulated wave equations are invariant.

Future work could be directed towards exploring SPCs of other types, as well as the more practical issue of the design and fabrication of polymeric materials that behave as per the suggested strain energy functions. For example, many soft rubbers such as silicone and neoprene [41] behave according to the neo-Hookean model when the stretch deformation is intermediate. A semilinear material could be envisaged as being realized in one dimension via, e.g. a linear spring-mass chain, but would certainly require significant efforts to synthesize in three dimensions. We anticipate that progress in polymer engineering is required to solve this challenge by designing polymers with the required microstructure. Additionally, it would be interesting to design SPCs with band gaps that are partially deformation-independent, namely, the band gaps are invariant for certain deformation modes or wave modes. Finally, it would seem appropriate for the sake of potential applications, to study other aspects of invariance of acoustic and elastic waves to deformation, whether induced by mechanical means or otherwise, e.g. electrical or magnetic.

## Data accessibility

There are no experimental data associated with this work. The simulation input files can be accessed through https://sites.google.com/site/puzhangsite/code.
